# Case series of dogs with blastomycosis managed with high-flow nasal oxygen therapy (2019–2023): 19 cases

**DOI:** 10.3389/fvets.2024.1369259

**Published:** 2024-06-05

**Authors:** Melanie Tarosky, Jenica L. Haraschak, Jennifer M. Reinhart, Audrey Billhymer

**Affiliations:** Department of Veterinary Clinical Medicine, University of Illinois Urbana-Champaign, Urbana, IL, United States

**Keywords:** acute hypoxemic respiratory failure, dogs, high flow nasal oxygen therapy, oxygen therapy, blastomycosis, systemic fungal infection

## Abstract

**Objective:**

To describe the clinical presentation, progression, treatment, and outcome of dogs with blastomycosis treated with high-flow nasal oxygen therapy (HFNOT).

**Design:**

Retrospective case review.

**Setting:**

University veterinary teaching hospital.

**Animals:**

Nineteen client-owned dogs with strongly suspected or confirmed blastomycosis treated with HFNOT.

**Measurements and main results:**

The medical records of dogs with strongly suspected or confirmed blastomycosis between October 2019 and May 2023 that received HFNOT were evaluated. Nineteen dogs were included. Nine dogs were started directly on high-flow nasal oxygen therapy. The remaining 10 dogs first received traditional oxygen therapy and were then transitioned to HFNOT 3–142 h later. Of the 19 dogs, 1 survived to discharge from hospital, 12 were euthanized due to progression of disease, and 6 died during the hospitalization period.

**Conclusions and clinical importance:**

The prognosis for survival of dogs with severe blastomycosis requiring therapy beyond traditional oxygen methods was poor to grave in this population. This is the first known documented report of HFNOT use in dogs with confirmed or suspected blastomycosis.

## Introduction

Blastomycosis is a severe, systemic fungal infection that affects dogs in the south central, upper midwestern, and southeastern United States. It has also been reported in the Canadian provinces which border the Great Lakes and St. Lawrence River, as well as India and Africa. The organisms exist as growing hyphae primarily in sandy soil rich with organic debris such as decaying vegetation and is released as airborne conidia by disruption of contaminated environments (1, 2). One or more organ systems may be involved, and pulmonary disease is common because inoculation occurs via conidia inhalation and the organism disseminates through the vascular or lymphatic system (3). Pulmonary signs occur in greater than 80% of affected dogs and are the most common reasons for presentation to a veterinarian (4). Other areas commonly affected include the skin, eyes, bones, reproductive tissues, and the central nervous system, which predictably lead to clinical signs based on site of infection. The severity of clinical signs can range from subclinical or self-limiting to severe clinical disease (5). The overall prognosis for dogs with confirmed *Blastomyces* infection is generally good (68–75%). However, dogs with severe lung disease have a lower survival rate of 30–60%; other negative prognostic indicators include central nervous system involvement and high band neutrophilia (6–9). In blastomycosis impacting the patient’s ability to oxygenate, the primary treatment, alongside anti-fungal medication, is oxygen therapy (10).

Traditional oxygen therapy (TOT), including cages, hoods, and nasal cannulation, is commonly used in canine pulmonary blastomycosis. However, these modalities are limited by patient tolerance, maximum flow rates, and the fraction of inspired oxygen (F_i_O_2_) that can be delivered (10). When TOT is inadequate to maintain appropriate arterial partial pressures of oxygen (P_a_O_2_) or carbon dioxide (P_a_CO_2_) or the patient is hemodynamically compromised or facing impending respiratory fatigue, more invasive techniques are indicated. Previously, the only method to achieve this was mechanical ventilation (MV), which requires specialized equipment, one-on-one staffing capabilities, anesthesia, and continuous monitoring and nursing care. Even at facilities in which this is feasible, it is often a cost-limiting and unattractive option to clients.

High-flow nasal oxygen therapy (HFNOT) represents an intermediate step between TOT and MV when escalating oxygen delivery. HFNOT provides heated and humidified medical gas allowing higher and adjustable flow rates, consistent F_i_O_2_ delivery up to 100%, dead space washout, and reduction of mild hypercapnia. The support is delivered to the patient by nasal cannulas, requiring mild to no sedation, which promotes patient tolerance of the apparatus. HFNOT also provides low-level positive end-expiratory pressure, which is theorized to improve pulmonary mechanics and reduce fatigue (11).

Since its first described use in veterinary patients with acute respiratory failure in 2016, HFNOT has increased in popularity as an alternative to TOT (12, 13). However, to the authors’ knowledge, the use of HFNOT in dogs with blastomycosis has not yet been reported. Given its reported success in other forms of acute hypoxemic respiratory disease, HFNOT could also benefit these cases (10, 11, 14). Therefore, the purpose of this study was to describe the clinical presentation, progression, treatment, and outcome of dogs with blastomycosis treated with HFNOT.

## Materials and methods

Case records were collected for client-owned dogs diagnosed with or suspected to have pulmonary blastomycosis and received HFNOT that were presented to the University of Illinois Veterinary Teaching Hospital (VTH) from October 2019 to May 2023. The search criteria within the electronic medical records were based on invoice charges found under “high flow” and “oxygen setup.” Dogs were included if (1) they were treated with HFNOT and (2) they had a diagnosis of blastomycosis confirmed on histology, cytology, serology, or urine antigen test or if blastomycosis was strongly suspected based on compatible clinical findings and exclusion of other diagnoses. In particular, respiratory signs (cough, tachypnea, dyspnea) in combination with a nodular interstitial or mixed thoracic radiographic pattern with or without perihilar lymphadenopathy was considered strongly suspicious for blastomycosis. Cases were excluded if the medical records were incomplete, if the patient had a primary diagnosis other than blastomycosis, or if the patient was escalated immediately to MV without use of HFNOT. The following data were abstracted from the medical record for each included case: signalment, clinical signs, physical examination findings (mentation, rectal temperature, heart rate, respiratory rate and effort, mucous membrane color, lung sounds, and abnormalities in other body systems), known organ systems affected by the fungal infection, comorbidities, clinicopathologic and radiographic findings, treatments including oxygen supplementation requirements, outcome, and total hospitalization cost.

**Figure 1:**
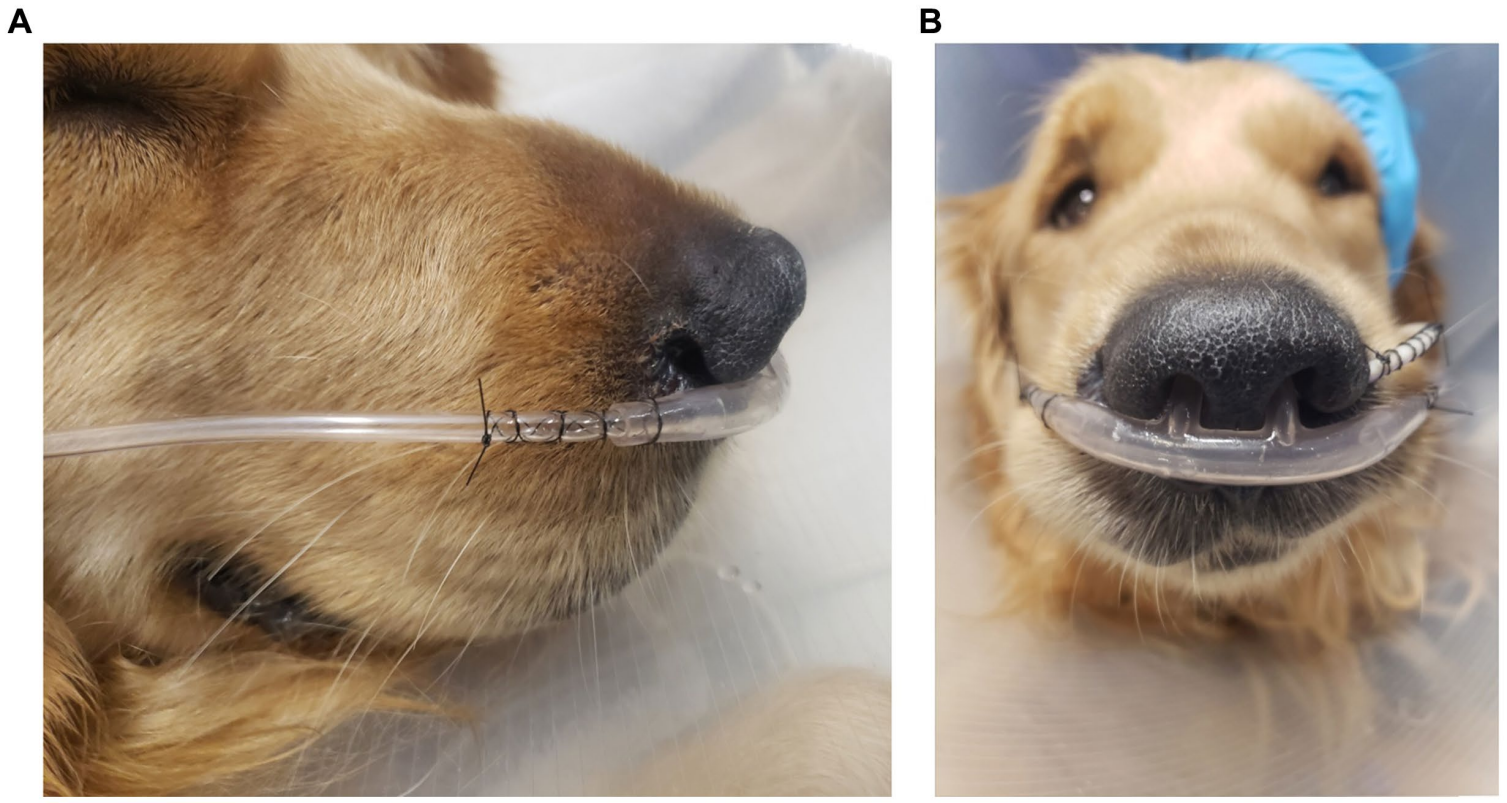
An example placement of patient nasal cannulas as demonstrated by lateral **(A)** and rostral **(B)** projections with finger trap pattern without tape bridge or tack sutures. Also pictured is a feeding tube secured similarly from the left nare.

For patients that received TOT, either a self-contained Intensive Care Unit by Snyder MFG was used as a cage unit, or a red rubber tube was placed as nasopharyngeal cannulation. These patients were initiated on 40% FiO2, or the 0.1–0.4 L/min flow rate of oxygen delivered to the nasopharyngeal cannula by a bubbler system. All patients were started on HNFOT using the Vapotherm Precision Flow System at 100% F_i_O_2_ and flow rate 1–2 L/kg/min after a 15–60-min 0.25–0.5 L/kg/min adjustment period. To facilitate oxygen delivery, bilateral disposable human nasal prongs were placed and sized to approximately 50% of the nare width as shown in Figure 1. Nasal prongs were secured with 3–0 or 2–0 nylon suture with a finger trap pattern on both sides. Either a tape bridge across the muzzle or tack sutures were used at the end of the finger trap pattern to further mold cannulas to the shape of the patient’s face. All patients received IV fluid therapy in addition to their medications. This study was exempt from institutional animal care and use committee approval.

All data are presented descriptively. The distributions of continuous data were assessed using the D’Agostino-Pearson and Kolmogorov–Smirnov normality tests (GraphPad Prism 10; San Diego, CA). Normally distributed data are presented as mean +/− standard deviation and non-normally distributed data are presented as median (range).

## Results

The medical records search yielded 48 records; 29 were excluded based on alternate diagnoses, and 19 dogs were included in the study. The breeds of the dogs within this population were Golden retriever (4/19, 21%), Labrador Retriever (4/19, 21%), mixed breed dog (4/19, 21%), Rottweiler (1/19, 5%), Hound (1/19, 5%), Great Dane (1/19, 5%), American Bulldog (1/19, 5%), German Shepherd (1/19, 5%), Beagle (1/19, 5%), and Shih Tzu (1/19, 5%). Three were intact males, 8 were male neutered, none were intact female, and 7 were female spayed. The mean (+/− standard deviation) age and weight were 3.7 +/− 2.3 years and 31 +/− 14 kg, respectively. Sixteen dogs had a clinical diagnosis of blastomycosis, some of which were diagnosed by more than one method. Ten dogs had positive lymph node cytology, 2 had positive skin lesion cytology, and 2 had positive lung cytology. Nine had a positive urine quantitative enzyme-linked immunoassay antigen test. One dog was diagnosed based on a fungal serology reported as positive and 1 was diagnosed on necropsy. Three dogs were presumptively diagnosed with blastomycosis based on a high level of clinical suspicion from presenting clinical signs, signalment, physical examination findings, and thoracic radiographs.

**Figure 2:**
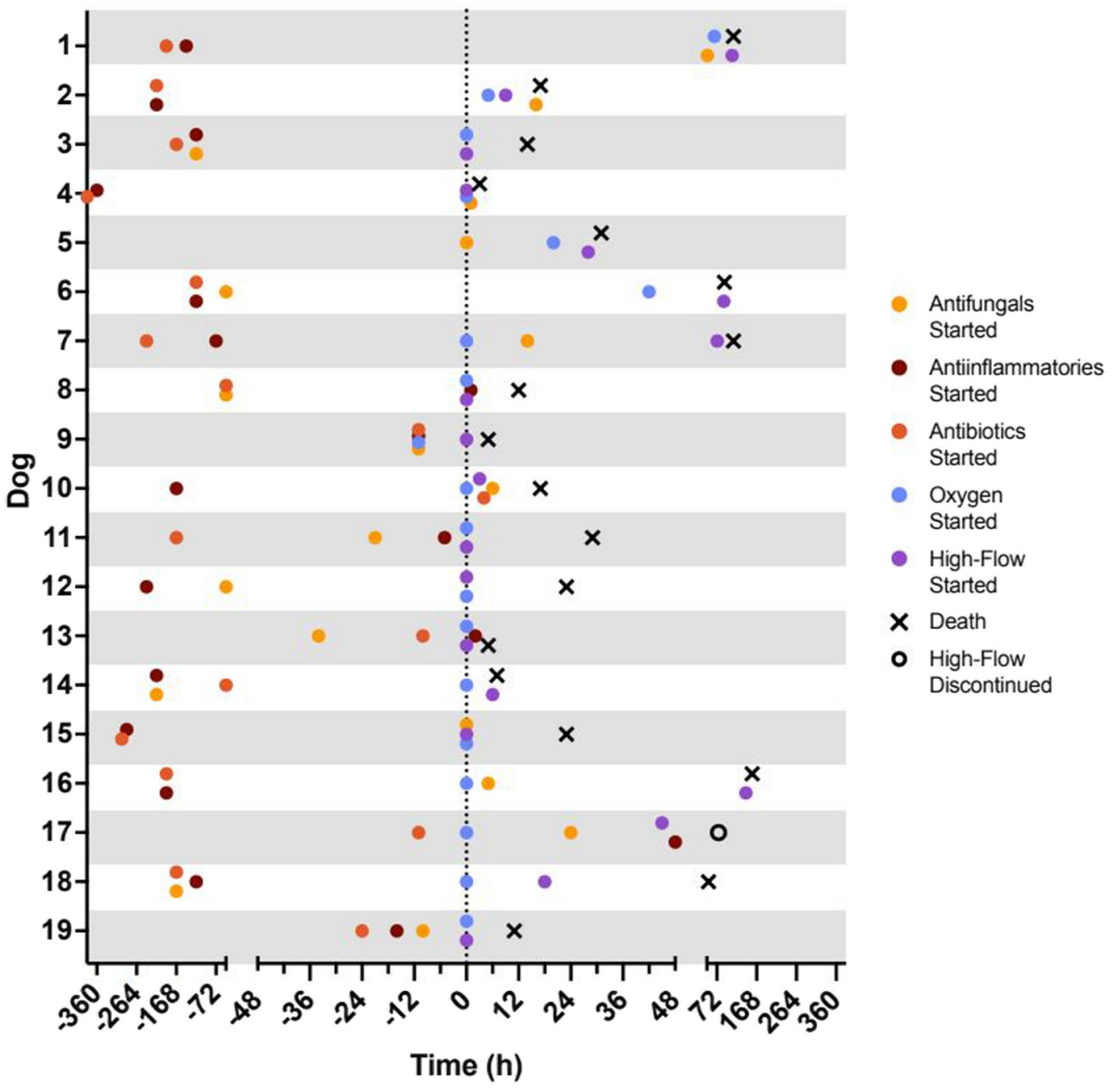
Timeline of treatment and outcomes for each patient. Time 0 h represents the time of admission to the VTH intensive care unit. Represented treatments include initiation of antifungal drugs (yellow circle), anti-inflammatory drugs (nonsteroidal anti-inflammatory drug or corticosteroid; red circle), antibiotics (green circle), traditional oxygen therapy (blue circle), and high-flow oxygen therapy (purple circle). Represented outcomes include successful discontinuation of high-flow oxygen therapy (open circle; *n* = 1) and death (black X; *n* = 18).

All dogs were presented through the VTH emergency service. Ten were directly referred from another veterinary facility, 8 had been seen previously by another veterinarian for related clinical signs but were not direct transfers, and 1 dog had not been evaluated previously. Prior to presentation, 17 dogs had received some form of medical therapy including 10 dogs that received at least one dose of an antifungal medication and 1 dog that received oxygen therapy (Figure 2). Three dogs had concurrent illnesses including 1 dog with hypothyroidism, 1 dog with controlled idiopathic epilepsy and hypothyroidism, and 1 dog with skin allergies.

Presenting complaints included constitutional signs (lethargy, anorexia) (17/19, 89%), tachypnea (17/19, 89%), dyspnea (16/19, 84%), lymphadenopathy (12/19, 63%), lameness or soft tissue swelling (11/19, 58%), skin lesions (8/19, 42%), gastrointestinal signs (7/19, 37%), coughing (5/19, 26%), ophthalmologic changes (4/19, 21%), and collapse (1/19, 5%). The median duration of respiratory signs prior to presentation was 4 days (0–30 days). The most common physical exam findings included increased respiratory effort (17/19, 89%), increased bronchovesicular or harsh lung sounds (16/19, 84%), tachypnea (15/19, 79%), fever (12/19, 63%), and pale or cyanotic mucous membranes (10/19, 53%). Point-of-care thoracic ultrasound findings were reported for 8 dogs and abnormalities included mild pleural effusion (2/19, 10%) and pulmonary parenchymal change (4/19, 21%). Only one patient had pulse oximetry performed with an oxygen saturation of 88%.

Arterial blood gas data at the time of intake were available for 5 patients. Two patients were evaluated on room air (F_i_O_2_ 21%). The first had a P_a_O_2_ of 46 mmHg and P_a_CO_2_ of 30.7 mmHg. The second, had a P_a_O_2_ of 67.7 mmHg and a P_a_CO_2_ 28.1 mmHg. One patient was evaluated while on TOT via cage unit (F_i_O_2_ 40%) with P_a_O_2_ of 80.1 mmHg and P_a_CO_2_ 25.2 mmHg. Two patients were evaluated while on HFNOT (F_i_O_2_ 100%); one with P_a_O_2_ 84 mmHg and P_a_CO_2_ 26.2 mmHg, the other with P_a_O_2_ 371.7 mmHg and P_a_CO_2_ not recorded. Venous blood gas findings were available for 13 patients and were largely unremarkable. Complete blood counts and serum biochemistry panels were available for 11 and 10 patients, respectively, and select parameters are summarized in Table 1. Leukocytosis with neutrophilia (6/11, 55%), left shift (4/11, 36%) and monocytosis (4/11, 36%) were the most frequent hematologic changes. Hyperphosphatemia (4/9, 44%) and hypoalbuminemia (9/9, 100%) were the most common biochemical alterations. In all patients that had a urinalysis performed (*n* = 10), proteinuria was identified (10/10, 100%). No other significant abnormalities were present on urinalysis.

**Table 1:** Select complete blood count and biochemistry parameters at admission.

Parameter	Unit	Reference interval	n	Mean +/− SD	Median (Range)	Normal distribution?
RBC	x10^6	5.5–8.5	11	5.6 +/− 1.3	5.3 (3.7–7.6)	Y
Hematocrit	%	35.52	11	37 +/− 8	37 (26–50)	Y
Hemoglobin	g/dL	12–18	11	13 +/− 2.9	13 (8.4–17)	Y
MCHC	g/dL	32.36	11	34 +/− 1.3	34 (32–37)	N
MCV	fL	60–77	11	67 +/− 3.7	68 (61–71)	Y
PLT	x10^3	200–700	11	285 +/− 125	276 (117–582)	N
WBC	x10^3	6–17	11	23 +/− 15	19 (5.4–62)	N
Neut	x10^3	3–11.5	11	19 +/− 15	16 (3.6–59)	N
Band	x10^3	0–0.3	11	0.6 +/− 1.4	0 (0–4.8)	N
Lymph	x10^3	1–4.8	11	1.2 +/− 0.79	1.1 (0.17–2.6)	N
Mono	x10^3	0.2–1.4	11	1.1 +/− 0.64	1.3 (0–1.9)	Y
Baso	x10^3	0–0.2	11	0.1 +/− 0.22	0 (0–0.74)	N
Eos	x10^3	0.1–1.0	11	0.22 +/− 0.37	0 (0–1.2)	N
Creatinine	mg/dL	0.5–1.5	9	0.84 +/− 0.47	0.7 (0.4–1.9)	N
BUN	mg/dL	6–30	10	25 +/− 24	17 (8–85)	N
TP	g/dL	5.1–7.0	9	6.1 +/− 0.78	6.1 (5.1–7.5)	Y
Albumin	g/dL	2.5–3.8	10	2.1 +/− 0.27	2.1 (1.7–2.6)	Y
Globulin	g/dL	2.7–4.4	9	4.1 +/− 0.61	4 (3.3–5.1)	Y
P	mg/dL	2.7–5.2	9	5.7 +/− 1.6	5 (4.4–8.4)	Y
Na	mmHg	141–152	9	146 +/− 5	146 (136–152)	Y
K	mmol	3.9–5.5	9	4.4 +/− 0.38	4.3 (3.9–4.9)	Y
Cl	mmol	107–118	9	113 +/− 4.4	114 (108–122)	Y
ALP	U/L	7–92	10	232 +/− 236	146 (27–808)	N
ALT	U/L	8–65	10	60 +/− 70	22 (12–230)	N
GGT	U/L	0–7	10	3.3 +/− 2.3	2.5 (0–7)	Y
Bilirubin	mg/dL	0–0.3	10	0.34 +/− 0.16	0.3 (0.2–0.6)	N

Thoracic radiographs were obtained in all patients; 5 dogs had radiographs performed only by a referring veterinarian, 9 dogs had radiographs performed only at the VTH, and 5 dogs had imaging performed at both locations. The radiographs for two patients could not be obtained from the referring hospitals and had no specialist review performed, and 1 patient record included a board-certified radiologist review, but images were not available. Referral case images were reviewed by a board-certified radiologist at the time of this report. Of the 17 radiology reports, 15 pulmonary parenchymal pattern distributions were described as diffuse (88%), 1 as focal (6%), and 1 as normal (6%). Six reports included a severity descriptor, of which 5 were labeled as severe (29%) and 1 as moderate (6%). The pulmonary parenchymal patterns consisted most commonly of a miliary pattern (5/17, 29%). The other patterns included mixed miliary with interstitial and/or alveolar changes (4/17 24%), mixed interstitial and alveolar (3/17, 18%), interstitial (2/17, 12%), alveolar (1/17, 6%), and normal (1, 6%). Eight patients, including the one with a normal pulmonary pattern, had intrathoracic, tracheobronchial lymphadenopathy (47%), 5 had pleural effusion (29%), and 3 had mild left atrial enlargement (18%). One dog had a cranial thoracic mass lesion of undetermined origin (6%).

The timelines for initiation of commonly used medications, oxygen therapy, and outcome are presented for each dog in Figure 2. During hospitalization, all dogs received intravenous fluid therapy while hospitalized. Five dogs received oral itraconazole with a median daily dose of 10 mg/kg (5–15 mg/kg). Eleven patients received fluconazole with a median daily dose of 10 mg/kg (8.6–16 mg/kg). Thirteen dogs were treated with dexamethasone at a median daily dose of 0.1 mg/kg (0.05–0.14 mg/kg); no dogs received nonsteroidal anti-inflammatory drugs while in hospital. All patients received some form of sedation to reduce anxiety related to dyspnea, 17 of which received butorphanol (89%). This was delivered as an intravenous bolus (0.2–0.4 mg/kg q 6 h) in 7 patients (37%) or as a constant rate infusion (0.1–0.4 mg/kg/h) in 9 patients (53%). Others received trazodone (6/19, 32%), dexmedetomidine (5/19, 26%), or acepromazine (5/19, 26%) as needed. Other medications administered included ondansetron (8/19, 42%), ampicillin/sulbactam (4/19, 21%), maropitant (4/19, 21%), pantoprazole (2/19, 10%), hydromorphone (2/19, 10%), amphotericin B, famotidine, capromorelin, gabapentin, fenbendazole, acetaminophen/codeine (1 dog, 5% each).

Fifteen dogs (78%) were started on oxygen therapy upon ICU admission and, of these, 9 (47%) were started directly on HFNOT with the remaining 6 (32%) transitioning from TOT to HFNOT 59 (3–142) hours later. For 4 dogs (21%), oxygen therapy was not started until 31 (5–66) hours after admission. All 4 dogs began with TOT and then transitioned to HFNOT 25.5 (4–47) hours later. The total time patients spent on the high-flow unit was 12 (1–39) hours (Figure 2).

Eighteen dogs died (6/19, 32%) or were euthanized due to clinical decline (12/19, 63%) during hospitalization. The median time to death or euthanasia was 20 (3–157) hours. One dog survived to discharge (dog 17, 1/19, 5%). This dog demonstrated tachypnea, dyspnea, lethargy, and hyporexia for 3 days prior to presentation. Increased bronchovesicular sounds and mild peripheral lymphadenopathy were present on physical exam, a focal alveolar pattern and pleural effusion were present on radiographs, and intralesional *B. dermatitidis* organisms were found on lung aspirate. No other organ systems appeared to be affected. This patient required TOT at the time of admission and escalation to HFNOT 45 h later. Despite being started at low initial flow rates of 0.25–0.5 L/kg/min, she was generally intolerant of the high-flow system, with frequent evidence of irritation such as sneezing, head shaking, pawing at face, and restlessness. Despite several attempts at adjusting sedation protocols, it was ultimately elected to discontinue her HFNOT after 31 h on the unit and continue TOT via nasal pharyngeal red rubber tube. She was weaned off oxygen to room air 63 h after discontinuation of HFNOT and discharged from hospital 147 h from the time of admission. For all dogs, the median cost of hospitalization was $2,336 ($921–$6,530).

## Discussion

HFNOT is a relatively new respiratory therapy in veterinary medicine and has multiple potential benefits over TOT (12, 13). It has reported success in various acute hypoxemic respiratory diseases of dogs (9, 11, 14). In the present study, 18/19 dogs (95%) with suspected or confirmed blastomycosis treated with HFNOT died or were euthanized. Thus, HFNOT may not perform as well in canine *Blastomyces* infections as other respiratory disease processes.

It is unclear why dogs in our study did not respond well to HFNOT compared to reports of other disease processes. Our study population was presented with severe respiratory disease as evidenced by the high proportions of tachypnea (17/19, 89%), dyspnea (16/19, 84%), and diffuse radiographic pulmonary change (15/17, 88%). These factors likely contributed to the decision to either escalate from TOT to HFNOT (10/19, 53%) or begin HFNOT at admission (9/10, 47%). The one dog that did survive had a similar clinical presentation to the non-survivors, but the radiographic distribution of its pulmonary lesions was focal rather than diffuse. The proportion of lung affected on radiographs has been demonstrated to predict survival in canine pulmonary blastomycosis (8). Thus, it is possible the severity of the lung lesions may also predict survival in dogs specifically on HFNOT, but more cases are needed before making that conclusion.

Additionally, there are several intrinsic features of blastomycosis that have been reported in people, which make the disease generally less responsive to oxygen therapy. Similar pathophysiologic mechanisms could exist in canine blastomycosis that might impact response to HFNOT. Blastomycosis, like other fungal diseases, is characterized by development of space occupying lesions in the pulmonary parenchyma (pyogranulomas), which severely impede lung function (3, 15). Decreased lung volume and compliance are characteristic of these diseases and have been documented in human tuberculosis, sarcoidosis, and histoplasmosis (16, 17). In advanced stages of the disease, significant interstitial pulmonary fibrosis may develop, which exacerbates hypoxemia through diffusion limitation, abnormalities of the pulmonary vasculature, and worsening ventilation-perfusion mismatch (18). Intrathoracic pyogranulomas and lymphadenopathy may also cause bronchial obstruction, resulting in lung lobe collapse, local occlusion or compression with subsequent pseudorestrictive pattern, and further worsening oxygenation and ventilation (17). In people, *Blastomyces* organisms stimulate a hyperinflammatory response and acute respiratory distress syndrome (ARDS) occurs in 10–15% of hospitalized blastomycosis patients (4, 15, 19). Despite intensive medical therapy, the mortality rate for these cases is 50–89%, which is significantly higher than the reported 42.9% mortality rate due to other causes of ARDS (4, 19–22). Finally, a die-off effect may contribute to disease severity in fungal infections, which results from temporary worsening clinical signs after initiation of antifungal therapy (23). It is unknown which of these mechanisms may be present in dogs with blastomycosis and to what extent. However, given the clinical similarity of canine blastomycosis to this disease in people, it is likely that some of these structural and functional respiratory changes contributed to the poor response to HFNOT seen in our study.

Pulmonary blastomycosis is a challenging disease to treat and definitive treatment protocols are not available for dogs, which may also have affected response to HFNOT in our study. In studies assessing response to different medical therapies, there was no significant difference found in survival for dogs receiving itraconazole versus fluconazole (24) and the role of anti-inflammatory drugs is controversial (25). The use of antibiotic therapy has not been defined, although concurrent bacterial pneumonia appears to be uncommon in dogs with pulmonary blastomycosis. In one study, 3 out of 94 dogs who had cytologic, histologic or bacterial culture performed on pulmonary samples had evidence of concurrent bacterial infection. Therefore, alveolar radiographic patterns or mixed patterns were more likely a result of the fungal infection and associated inflammation or other pulmonary disease process, rather than secondary infection (9). Thus, the treatment regimen that yields maximal efficacy for canine blastomycosis has not been established.

Supplemental oxygen is considered an important supportive measure in dogs with pulmonary blastomycosis causing hypoxemia. Options include TOT, MV, and HFNOT. TOT is commonly used in these patients because it is easy and relatively accessible, although its impact on survival is unknown. The use of MV has also been described in canine blastomycosis but with poor results. In one study which used MV to treat 5 dogs diagnosed with pulmonary blastomycosis, none survived (9). Although HFNOT did not appear to perform well for dogs with blastomycosis in our study, published veterinary reports suggest that HFNOT may be helpful in other diseases associated with respiratory failure as a bridge between TOT methods and MV. In one of these prior report, HFNOT improved oxygenation as well as reduced the work of breathing in dogs who required escalation from traditional oxygen therapy. These patients were described as having acute hypoxic respiratory failure, though the specific diagnoses were not listed (26). This conclusion was similar to that of Frischer, et al. (11), who found that initiation of HFNOT in dogs with respiratory disease significantly improved P_a_O_2_, blood oxygen saturation, and respiratory rate. Even in the absence of primary lower airway disease, improvement in respiratory parameters was seen when this modality was used in dogs with brachycephalic obstructive airway syndrome recovering from general anesthesia (26).

In this case series, dogs with blastomycosis treated with HFNOT had a poor survival rate (5%). However, HFNOT may still have value in some cases, possibly if instituted sooner in the clinical course of disease. Evaluation of a larger group of dogs may find a subpopulation that is most responsive to this treatment. Further studies are warranted to investigate factors which may affect response to HFNOT including severity of respiratory dysfunction and time of initiation of oxygen support. Low-flow strategies and combination treatments should also be evaluated.

This study was limited by its retrospective nature. Although the general set-up for HFNOT was the same for all patients, timing of initiation and protocol varied. TOT and HFNOT flow rates were not consistently documented and so could not be included, and arterial blood gas analyses were not performed routinely for all patients. Most patients did not have notations as to whether HFNOT-related complications occurred such as excessive aerophagia, poor tolerance to the nasal cannulas, or poor tolerance of high flow rates. All patients had varied sedation strategies based on clinician preference. Similarly, nearly all patients received both anti-fungal and anti-inflammatory therapies, but the specific drugs, doses and times of initiation were variable. This study only included dogs that actually received HFNOT; patients that needed advanced oxygen support but declined care or were euthanized prior to initiation were not included due to limitations of the electronic medical records search. Similarly, it would have been very interesting to compare dogs with known or suspected severe pulmonary blastomycosis that did and did not receive HFNOT, but the search methodology precluded such a comparison.

To the authors’ knowledge this is the first report describing HFNOT use in dogs with known or suspected severe pulmonary blastomycosis. The overall outcome was poor with 1/19 (6%) dogs surviving to discharge. However, these results likely reflect the severity of disease and structural and functional lung pathology associated with blastomycosis rather than an innate failure of the treatment. Thus, HFNOT may still be useful in a subset of patients. Future studies should aim to identify dogs most likely to benefit from this therapy and strategies to improve outcomes.

## Data availability statement

The original contributions presented in the study are included in the article/supplementary material, further inquiries can be directed to the corresponding author.

## Ethics statement

Ethical approval was not required for the studies involving animals in accordance with the local legislation and institutional requirements because this was a retrospective evaluation and was exempt from institutional animal care and use committee approval. Written informed consent was not obtained from the owners for the participation of their animals in this study because this was a retrospective evaluation, and all data was extracted from medical records. There was no use of any client-owned animals or samples from client-owned animals in this study.

## Author contributions

MT: Conceptualization, Data curation, Investigation, Methodology, Project administration, Resources, Visualization, Writing – original draft, Writing – review & editing. JH: Conceptualization, Data curation, Methodology, Supervision, Writing – review & editing, Investigation. JR: Data curation, Formal analysis, Investigation, Resources, Supervision, Validation, Visualization, Writing – review & editing. AB: Data curation, Formal analysis, Investigation, Writing – review & editing.

## References

[1] Kerl ME. Update on canine and feline fungal diseases. Vet Clin North Am Small Anim Pract. (2003) 33:721–47. doi: 10.1016/S0195-5616(03)00035-4, PMID: 12910740

[2] Sykes JE. Canine and feline infectious diseases. St. Louis, Mo: Elsevier/Saunders (2014).

[3] Bromel C Sykes JE. Epidemiology, diagnosis, and treatment of blastomycosis in dogs and cats. Clin Tech Small Anim Pract. (2005) 20:233–9. doi: 10.1053/j.ctsap.2005.07.004, PMID: 16317913

[4] Shelnutt LM Kaneene JB Carneiro PAM Langlois DK. Prevalence, distribution, and risk factors for canine blastomycosis in Michigan, USA. Med Mycol. (2020) 58:609–16. doi: 10.1093/mmy/myz110, PMID: 31732747 PMC7326585

[5] Herrmann JA Kostiuk SL Dworkin MS Johnson YJ. Temporal and spatial distribution of blastomycosis cases among humans and dogs in Illinois (2001-2007). JAVA. (2011) 239:335–43. doi: 10.2460/javma.239.3.335 21801047

[6] Legendre AM Rohrbach BW Toal RL Rinaldi MG Grace LL Jones JB. Treatment of blastomycosis with itraconazole in 112 dogs. J Veterin Intern Med. (1996) 6:365–71. doi: 10.1111/j.1939-1676.1996.tb02082.x, PMID: 8947868

[7] Legendre AM Selcer BA Edwards DF Stevens R. Treatment of canine blastomycosis with amphotericin B and ketoconazole. J Am Vet Med Assoc. (1984) 184:1249–54. 6330013

[8] Motschenbacher LO Furrow E Rendahl AK Nell EG Anderson KL Merkel LK. Retrospective analysis of the effects of Blastomyces antigen concentration in urine and radiographic findings on survival in dogs with blastomycosis. J Vet Intern Med. (2021) 35:946–53. doi: 10.1111/jvim.16041, PMID: 33604957 PMC7995372

[9] Crews LJ Feeney DA Jessen CR Newman AB Sharkey LC. Utility of diagnostic tests for and medical treatment of pulmonary blastomycosis in dogs: 125 (1989-2006). J Am Vet Med Assoc. (2008) 232:227–7. doi: 10.2460/javma.232.2.222 18275389

[10] Keir I Daly J Haggerty J Guenther C. Retrospective evaluation of the effect of high flow oxygen therapy delivered by nasal cannula on PaO2 in dogs with moderate-to-severe hypoxemia. J Vet Emerg Crit Care. (2016) 26:598–602. doi: 10.1111/vec.12495 27333466

[11] Frischer R Daly J Haggerty J Guenther C. High-flow nasal cannula improves hypoxemia in dogs failing conventional oxygen therapy. J Am Vet Med Assoc. (2023) 261:210–6. doi: 10.2460/javma.22.09.0400 36322486

[12] Krawec P Marshall K Odunayo A. A review of high flow nasal cannula oxygen therapy in human and veterinary medicine. Top Companion Anim Med. (2022) 46:100596. doi: 10.1016/j.tcam.2021.100596 34757156

[13] Whitney J Keir I. Clinical review of high-flow nasal oxygen therapy in human and veterinary patients. Front Vet Sci. (2023) 10:1070881. doi: 10.3389/fvets.2023.1070881, PMID: 36950541 PMC10027015

[14] Hopper K Powell LL. Advanced oxygen therapy for the small animal patient - high-flow oxygen therapy and mechanical ventilation. Vet Clin North Am Small Anim Pract. (2022) 52:689–705. doi: 10.1016/j.cvsm.2022.01.006, PMID: 35379497

[15] Romani L. Cell mediated immunity to fungi: a reassessment. Med Mycol. (2008) 46:515–29. doi: 10.1080/13693780801971450 19180748

[16] Stanescu DC Teculescu DB Racoveanu C. Lung function in acute pulmonary histoplasmosis. Chest. (1971) 60:105–7. doi: 10.1378/chest.60.1.105, PMID: 5571265

[17] West JB Luks AM. West's pulmonary pathophysiology. Philadelphia, PA: Wolters Kluwer (2017).

[18] Johannson KA Pendharkar SR Mathison K Fell CD Guenette JA Kalluri M. Supplemental oxygen in interstitial lung disease: an art in need of science. Ann Am Thorac Soc. (2017) 14:1373–7. doi: 10.1513/AnnalsATS.201702-137OI, PMID: 28644693

[19] Meyer KC McManus EJ Maki DG. Overwhelming pulmonary blastomycosis associated with the adult respiratory distress syndrome. N Engl J Med. (1993) 329:1231–6. doi: 10.1056/NEJM199310213291704, PMID: 8413389

[20] Bellani G Laffey JG Pham T Fan E Brochard L Esteban A. Epidemiology, patterns of care, and mortality for patients with acute respiratory distress syndrome in intensive care units in 50 countries. JAMA. (2005) 315:788–800. doi: 10.1001/jama.2016.0291 26903337

[21] Lemos LB Baliga M Guo M. Acute respiratory distress syndrome and blastomycosis: presentation of nine cases and review of the literature. Ann Diagn Pathol. (2001) 5:1–9. doi: 10.1053/adpa.2001.21473, PMID: 11172200

[22] Vasquez JE Mehta JB Agrawal R Sarubbi FA. Blastomycosis in Northeast Tennessee. Chest. (1998) 114:436–43. doi: 10.1378/chest.114.2.436, PMID: 9726727

[23] Jaiswal N Kumar A. Candida die-off: adverse effect and neutralization with phytotherapy approaches. Toxicon. (2024) 237:107555. doi: 10.1016/j.toxicon.2023.107555, PMID: 38072320

[24] Mazepa AS Trepanier LA Foy DS. Retrospective comparison of the efficacy of fluconazole or itraconazole for the treatment of systemic blastomycosis in dogs. J Vet Intern Med. (2011) 25:440–5. doi: 10.1111/j.1939-1676.2011.0710.x, PMID: 21418325

[25] Walton RAL Wey A Hall KE. A retrospective study of anti-inflammatory use in dogs with pulmonary blastomycosis: 139 cases (2002-2012). J Vet Emerg Crit Care. (2017) 27:439–43. doi: 10.1111/vec.12615, PMID: 28561957

[26] Jagodich TA Bersenas AME Bateman SW Kerr CL. High-flow nasal cannula oxygen therapy in acute hypoxemic respiratory failure in 22 dogs requiring oxygen support escalation. J Vet Emerg Crit Care. (2020) 30:364–75. doi: 10.1111/vec.12970, PMID: 32583614

